# Early life adoption shows rearing environment supersedes transgenerational effects of paternal stress on aggressive temperament in the offspring

**DOI:** 10.1038/s41398-021-01659-2

**Published:** 2021-10-16

**Authors:** Ipshita Zutshi, Sonakshi Gupta, Olivia Zanoletti, Carmen Sandi, Guillaume L. Poirier

**Affiliations:** 1 Laboratory of Behavioral Genetics, Brain Mind Institute, École Polytechnique Fédérale de Lausanne (EPFL), CH-1015 Lausanne, Switzerland; 2 Neuroscience Institute and Department of Neurology, Langone Medical Center, New York University, New York, NY USA; 3 Pharmacy Department, Birla Institute of Technology & Science Pilani, Hyderabad Campus, Hyderabad, India

**Keywords:** Human behaviour, Epigenetics and behaviour

## Abstract

Prenatal experience and transgenerational influences are increasingly recognized as critical for defining the socio-emotional system, through the development of social competences and of their underlying neural circuitries. Here, we used an established rat model of social stress resulting from male partner aggression induced by peripubertal (P28-42) exposure to unpredictable fearful experiences. Using this model, we aimed to first, characterize adult emotionality in terms of the breadth of the socio-emotional symptoms and second, to determine the relative impact of prenatal vs postnatal influences. For this purpose, male offspring of pairs comprising a control or a peripubertally stressed male were cross-fostered at birth and tested at adulthood on a series of socio-emotional tests. In the offspring of peripubertally stressed males, the expected antisocial phenotype was observed, as manifested by increased aggression towards a female partner and a threatening intruder, accompanied by lower sociability. This negative outcome was yet accompanied by better social memory as well as enhanced active coping, based on more swimming and longer latency to immobility in the forced swim test, and less immobility in the shock probe test. Furthermore, the cross-fostering manipulation revealed that these adult behaviors were largely influenced by the post- but not the prenatal environment, an observation contrasting with both pre- and postnatal effects on attacks during juvenile play behavior. Adult aggression, other active coping behaviors, and social memory were determined by the predominance at this developmental stage of postnatal over prenatal influences. Together, our data highlight the relative persistence of early life influences.

## Introduction

Increasing evidence supports the importance of nongenomic transgenerational transmission for the programming of physiological and/or behavioral traits [1–5]. Stress is considered to be one of the strongest modulatory factors for this nongenomic transmission across generations i.e., from stress in the parents (father, mother, or both) to the offspring [6–9].

Over the years, both clinical and preclinical studies have shown transmission of aggression from parents to their offspring, particularly in the context of highly stressful interpersonal partner violence. However, in most of these studies, the mothers/dams (the victims of violence) raising the offspring had shown depression-like symptoms which might have contributed to the behavioral changes observed in the offspring [10–17]. Furthermore, postnatal interactions between mother and her offspring during early life periods are known to impact the stress response (behavioral as well as hormonal) of these offspring in adulthood [18]. Nevertheless, gaps still exist in the literature addressing the effects of various postnatal interactions, specifically with regards to mammals [19–21].

Thus, a key question that needs to be addressed is to what extent postnatal factors contribute to the behavioral alterations observed in the offspring of stressed parents. While drawing this distinction poses difficulties in humans, it is, to some extent, possible to disentangle the prenatal factors from that of postnatal through animal studies. To this end, our lab has reported an animal model (aggression induced by peripubertal stress, PPS) to study transgenerational transmission of intimate partner violence in which the offspring of the aggressive male rats, which are raised by the victimized dams, also exhibit increased aggression including aberrant aggression against their female partners [22–25].

Here, to further our understanding of the factors contributing to such transgenerationally induced behavioral alterations in offspring, we investigated whether postnatal maternal factors play a role in the level of aggression and associated behavioral phenotypes in the offspring. Specifically, we cross-fostered the male offspring into four different groups after birth. Cross-fostering of offspring has been previously adopted by many research groups to successfully study the impact of maternal environmental factors (both prenatal and postnatal) on the behavioral development of offspring (reviewed in [26]). This paradigm helps by segregating the prenatal factors, i.e., gestational influences, from that of postnatal, i.e., alterations in maternal behavior which were induced by exposure to an aggressive partner. Cross-fostering that lasted from birth till weaning of the pups allowed us to examine the ways in which prenatal programming gets influenced by postnatal environmental factors. Notably, it also helped us study the development of resilience in offspring.

Considering mechanisms of transgenerational transmission, the present cross-fostering study revealed a potential developmental distinction for antisocial behaviors, whereby both pre- and postnatal effects were observed in juveniles, but postnatal influences predominated at adulthood.

## Materials and methods

### Animals

The experimental subjects were in-house bred offspring of Wistar Han rats (Charles River Laboratories, L’Arbresle, France), housed in a standard plastic cage on a 12-h light-dark cycle (lights on at 4:00 am). Food and water were available ad libitum. All procedures were conducted in conformity with the Swiss National Institutional Guidelines on Animal Experimentation and approved by a license from the Swiss Cantonal Veterinary Office Committee for Animal Experimentation. Paternal condition was determined by exposure to control or peripubertal stress conditions throughout P28-42, according to a previously detailed protocol [23–25, 27]. Briefly, unpredictable stress was experienced following a regimen comprising of exposure (25 min) to an elevated platform or predator odor, either exclusively (P34, P36, P42) or sequentially (P28-30, P40). The control animals were handled on the days that their experimental counterparts were exposed to stress. Due to the challenging nature of maintaining four different groups of cross-fostered animals over two generations, pilot data could not be collected to perform a power analysis to determine the sample size. However, the sample size (*n* = 32, 8 per group) is comparable to that used in previous studies [23].

### Experimental design

**Fig. 1:**
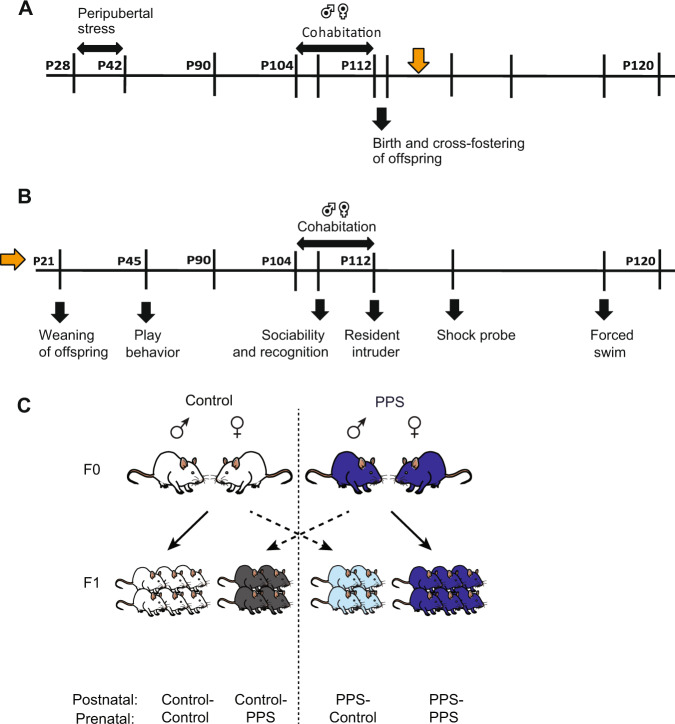
Schematic of the experimental procedure and cross-fostering of pups. **A** Timeline of experimental procedure on F0 generation males and cross-fostering of pups. Orange arrow, start of behavioral experiments on F1 generation. See Supplementary figure 1 for a detailed description of the behavioral tests performed on F0 males. **B** Experimental timeline of behavioral tests performed on the F1 males. **C** For cross-fostering, the pups were divided into four groups—(i) Control–Control - born to and raised by the same dam, paired with a control male, (ii) Control-PPS—born to a dam paired with a PPS male, but fostered with a dam paired with a control male, (iii) PPS-Control—born to a dam paired with a control male, but fostered with a dam paired with a PPS male and (iv) PPS–PPS—born to, and raised by the same dam, paired with a PPS male.

Births began three days after the parental cohabitation was ended by removing the F0 male (Fig. 1A). Litters comprising eight or more pups were used for cross-fostering, and where necessary culling to ten pups was applied. On day 1 of birth (at 11 am each day), pups were weighed and 4 pups from each litter of a group were exchanged. At the time of cross-fostering we were not aware of the sex of the pups, and therefore cross-fostered 4 pups at random. Each pup was tattooed by green tattoo paste (24201-01, Fine Science Tools GmbH, Germany) using Micro Tattoo System (24201-00, Fine Science Tools GmbH, Germany) on either its left front paw (for adoptees) or its right front paw (for biological offspring). All the pups were rolled in the bedding and sprinkled with sawdust to help minimize the potential identification of the adoptees by the dams, a practice previously shown to be sufficient to preclude any potential differences in maternal behavior [28]. Unpaired litters, or those with a low litter size, were sham fostered, by marking all the pups with the tattoo and returning them to their birth mother. The pups were weaned on day 21, and were housed with unfamiliar males with matched weights, belonging to the same testing group. At the time of weaning, the F1 females (either cross-fostered or not) were sacrificed, and the F1 males were subsequently classified into four groups (Fig. 1B)—Control–Control (born to and raised by the same dam, paired with a control male), Control-PPS (born to a dam paired with a PPS male, but fostered with a dam paired with a control male), PPS-Control (born to a dam paired with a control male, but fostered with a dam paired with a PPS male) and PPS-PPS (born to, and raised by the same dam, paired with a PPS male). They were housed two animals per standard plastic cage and following cohabitation with adult females at P90 for 21 days, the males were singly housed for 7–8 weeks before sacrificing, during which a battery of tests was performed (Fig. 1C). Both F0 and F1 males underwent the same set of behavioral tests (Fig. 1A, B, Supplementary Fig. 1, Supplementary Fig. 2). In addition, dams paired with these F0 males also underwent additional behavior testing (Supplementary Methods, and Supplementary Figs. 3–5).

### Behavioral testing

#### Play behavior

**Fig. 2:**
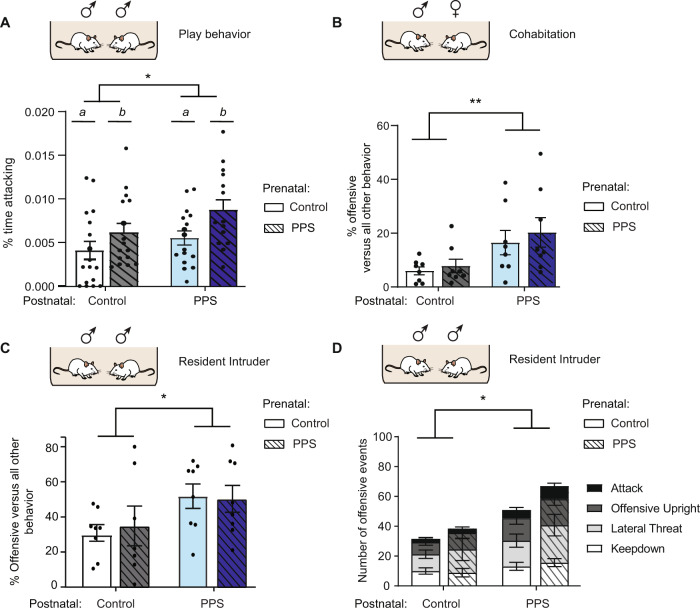
Postnatal environment explains aggressive tendencies in F1 males. **A** Play behavior. In juveniles, aggressive temperament was enhanced during home cage play behavior as a result of both prenatal and postnatal PPS dam influences. Prenatal effects are depicted by letters a, b. **B** Cohabitation. Only the adults reared by PPS male-paired dams exhibited increased aggressive behavior towards females during cohabitation. **C** Resident-intruder test. Adults reared by PPS male-paired dams exhibited increased aggressive behavior towards an intruder male as evidenced through increased proportion of offensive behaviors compared to all other behaviors. **D** Increased aggression in males reared by PPS male-paired dams was also measured in terms of the number of offensive events. The results are the mean ± s.e.m. PPS: Peripubertally stressed rats. * P < 0.05, ** P < 0.01.

The play behavior was performed on F1 male Wistar rats (Charles River), as previously reported [29]. On P44, rats were made to individually habituate to their test cages for a period of 10 min. In order to enhance their social motivation, the animals were socially isolated for a period of 3.5 h before testing on P45 [30]. In the test cage, two animals from the same experimental group (body weight difference not exceeding 10 g) were caged together for 15 min. The interaction was recorded by placing the cage in front of a camera. A trained experimenter blind to experimental groups analyzed the following behaviors using the software, Observer (Noldus IT; Netherlands)—pinning (i.e. keeping down), attacking, or pouncing (i.e. rubbing the neck), kicking, biting, and social exploration. The cumulative duration of attacking, keeping down, kicking, and biting behavior was calculated as the Total Aggressive Behavior (TAB) [29]. The F1 males were divided randomly into two cohorts, and both cohorts were examined for differences in play behavior (Fig. 2). For all subsequent tests, however, only one cohort was examined, while the other cohort was sacrificed to test for differences in blood and brain samples. These biological data are not included in this manuscript.

#### Cohabitation

Adult (10-week old, *N* = 24, 12 animals for each group) virgin female Wistar rats (Charles River Laboratories) were housed upon arrival in groups of three per cage. Two weeks later, they were housed with a 3-month-old adult male (either control or peripubertally stressed/PPS) for 18 days. The weight and anxiety of the females were balanced between the control and peripubertally stressed groups of males, including with pair matching by equivalent anxiety-like rank (% time in open arm of EPM), in light of our prior finding that this female behavioral tendency can have an impact on their reactivity to the male partner behavior and is associated with a subsequent depressive-like outcome [25]. At the end of this cohabitation period, the male was removed before parturition and tissue paper was provided to facilitate nest building. Following weaning, the dams remained singly housed in the same breeding cage for 15 days. Behavioral testing was performed 5 days after weaning (Fig. 1C). The results for only those dams which gave birth were included during analysis.

During male–female cohabitation, immediately after each male and female were put together in a cage, the rats were video-recorded for 30 min. The number of attacks from the male to the female, including mounting, chasing, keep down, offensive upright and lateral threat) and the time during which the female displayed defensive-submissive behavior (either freezing, kicking or being in a supine position under the male) were quantified, assisted by a computer program (Clicker V1.13, Velibor ilic, 2005).

In addition, the F1 males were also paired with adult virgin female Wistar rats (10-week old, *N* = 32, 8 animals for each group), and the same behavior paradigm was repeated in this generation.

#### Resident intruder (RI)

Aggression towards another male was investigated using a resident-intruder test [24], adapted from [31] Veenema et al (2006). Each rat was housed in an experimental cage (40 × 29 × 20 cm) with a female Wistar rat for 10 days. The RI tests were conducted during the beginning of the dark cycle, when the female was removed from the resident’s home‐cage 5 min before each test and was returned after the test. During the standard RI test, the resident control or peripubertally stressed male was exposed in its home‐cage for 30 min to an unfamiliar male Wistar Han rat (used only once), of approximately equivalent weight and anxiety-like tendencies (±5% difference in open arm of EPM) and identified with stripes all over its body by a black marker). The tests were video-recorded, and the behavioral scoring was conducted assisted by a computer program (The Observer 5.0.25, Noldus, 2003). The following standard parameters related to intermale aggression were scored for the intruders and the residents: attack latency time, number of attacks, lateral threat, offensive upright, and keep down. The percentage of the time spent performing the latter three behavioral parameters was summarized as the total aggressive behavior. Furthermore, social behavior such as hetero-grooming and mounting, self‐grooming and the submissive behaviors of the intruders and residents were also scored. Aggression by the resident was quantified by calculating (total aggressive behavior*100/total aggressive behavior + hetero-grooming by the resident + sniffing by the resident). This test was performed on both F0 and F1 males, and the data for both generations are included (Fig. 2, Supplementary Fig. 2).

#### Social investigation

##### Sociability

**Fig. 3:**
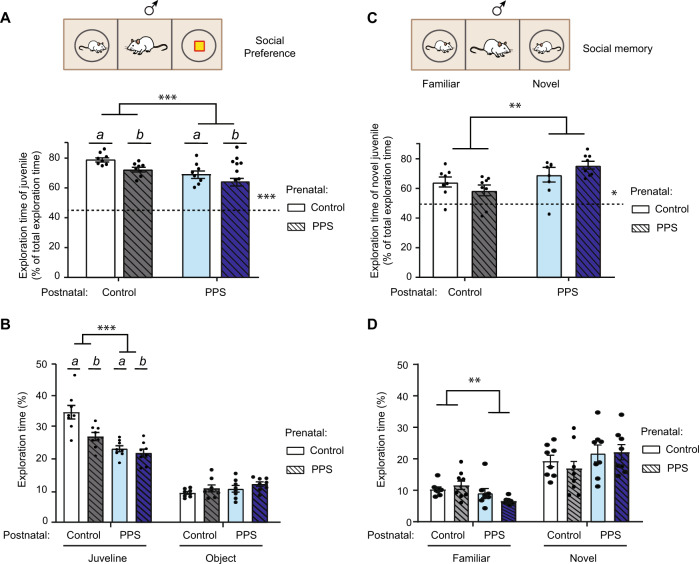
Offspring of PPS males show a lower inclination for social contact. **A** Social Preference. The exploration percentage (expressed as the time spent exploring the juvenile over total exploration) was significantly reduced both by being born to or reared by PPS male-paired dams. Prenatal effects are depicted by letters a, b. **B** The total time spent exploring the juvenile was significantly reduced by both prenatal and postnatal effects, while there was no effect on object exploration time. Prenatal effects are depicted by letters a, b. **C** Social memory. Social memory was enhanced in pups raised by PPS male-paired dams as evident by greater novel juvenile exploration percentage when compared to total exploration time. **D** The effect in social memory was driven by decreased exploration of the familiar juvenile by offspring raised by PPS male-paired dams, with no changes in exploration of the novel juvenile. n = 8 per group. The results are the mean ± s.e.m. PPS: Peripubertally stressed rats. * P < 0.05, ** P < 0.01, *** P < 0.001.

Social tendencies were evaluated by contrasting free access to a non-threatening juvenile or an inanimate object [24]; adapted from Crawley and collaborators [32], and as previously described [33]. Briefly, the test was conducted in a rectangular, three-chambered apparatus fabricated from gray opaque polycarbonate, with each dividing wall comprising a retractable door. In each lateral, target area was a transparent, perforated Plexiglass cylinder (15 cm diameter) receiving either the social (unfamiliar male juvenile rat approximately 34 days old) or non-social stimulus (yellow plastic bottle). The juvenile rats were first habituated to the three-chambered apparatus by placing them individually in the box within a cylinder for 10 min during the 3 days preceding the test. On the test day, an experimental rat was introduced into the closed off central chamber and after 5 min the social and inanimate cues were placed in either adjacent compartment, in a counterbalanced position across sessions, whence both doors to the side chambers were carefully removed, opening up the entire apparatus for a 10-min video-recorded exploration session. This test was performed on both F0 and F1 males, but only the data for the F1 generation are included (Fig. 3).

##### Social memory

Immediately after the sociability test, the rats were returned to their home cage for 30 min, as previously reported [34]. After this, using the same apparatus and protocol as the preceding sociability test, the social memory test paradigm consisted of 10-min exploration trials. An initial trial was conducted by returning the first juvenile to the same side of the chamber and placing a novel juvenile instead of the non-social stimulus. Following this, the test rats were again returned to their home cages for 30 min. The procedure was repeated a third time, where no confounding switch from inanimate to social target was made, each target (novel or familiar) being social, and only the results from this trial are thus considered. The second juvenile remained the same, but the first juvenile was replaced with a novel, third one. The testing duration was maintained as 10 min again.

The apparatus was cleaned with 5% ethanol and dried thoroughly between each test. The time spent sniffing each cylinder [35] was manually scored using Clicker 1.13 (Velibor ilic, 2005) by an experimenter blind to the treatments to evaluate the level of preference for the unfamiliar juvenile compared with the object or the familiar juvenile (exploration time ratio = time exploring the (unfamiliar) juvenile/time exploring the object or familiar juvenile; exploration time % = time exploring the (unfamiliar) juvenile/total time exploring both the object and juvenile).

This test was performed on both F0 and F1 males, but only the data for the F1 generation are included (Fig. 3).

#### Shock probe

One week after the end of the cohabitation, a test of active coping was conducted, adapted from [36] Pinel and Treit (1978). A rat was placed in a Plexiglas chamber with approximately 2 inches of bedding material (sawdust). A wire-wrapped prod/probe (Ø = 1 cm; 6–7 cm long) inserted through a small hole 2 cm above the bedding in one of the test chamber walls upon contact transmitted a 0.6 mA current. The shock was active throughout the whole experiment and each session was timed for 15 min after the first shock. Rat behavior was video-recorded, and time spent either away, or towards the probe, latency to start burying, percentage of time spent burying, probe explore, grooming, rearing, and immobility were manually quantified with the aid of a computer program (Clicker V1.13, Velibor ilic, 2005).

**Fig. 4:**
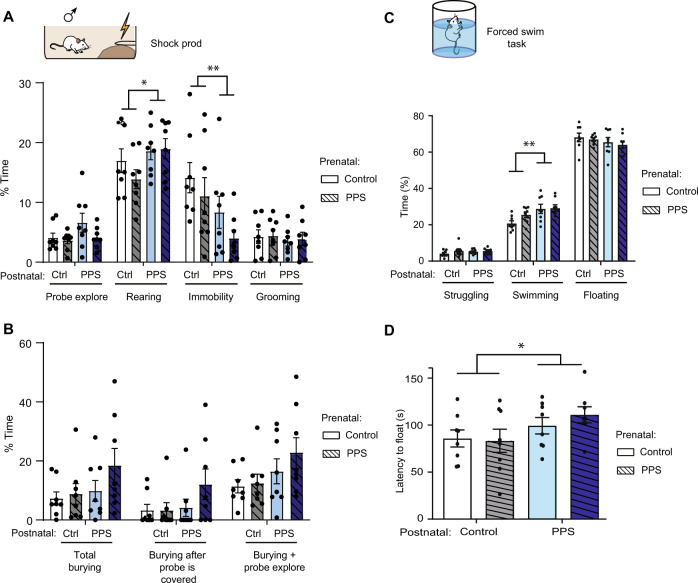
Adults raised by PPS male-paired dams display enhanced coping behaviors. **A** Shock probe, passive coping—the pups born to or raised by PPS male-paired dams exhibited less immobility in response to the shock and increased rearing. **B** Shock probe, active coping—no significant difference was observed in pups reared by PPS male-paired dams in terms of probe burying or probe exploration. **C** Forced swim test. The pups raised by PPS male-paired dams showed more active coping by a postnatal increase in the time spent swimming. **D** The increase in swimming duration was accompanied by a postnatal effect on the latency to float. *n* = 8 per group. The results are the mean ± s.e.m. Ctrl: Control, PPS: Peripubertally stressed rats. * P < 0.05, ** P < 0.01, *** P < 0.001.

This test was performed on both F0 and F1 males, but only the data for the F1 generation are included (Fig. 4).

#### Forced swim

The rats were submitted to a forced swim test to evaluate depression‐like behavior [37]. The animals were individually placed in a plastic beaker (25 cm diameter, 46 cm deep) containing 30 cm of water (25 °C) for 15 min (6 or 4 animals at a time). A second session was performed 24 h later for 5 min. Their behavior was recorded with a video camera, and the time spent immobile (making only those movements necessary to keep the snout above the water), swimming, climbing or diving was manually quantified with the aid of a computer program (Clicker V1.13, Velibor ilic, 2005). The rats were dried thoroughly before being returned to their home cages.

This test was performed on both F0 and F1 males, and the data for both generations are included (Fig. 4, Supplementary Fig. 2).

#### Statistics

Statistical analyses were performed with Statistics Package for the Social Sciences (SPSS; Zürich, Switzerland). Normality and homogeneity of variance were tested, and adjusted statistics were used accordingly. Parametric statistics were applied when data were normally distributed according to the Shapiro–Wilk test. Mean comparisons were carried out with either Student’s *t* tests (or Wicoxon signed-rank tests where indicated) or analyses of variance (either one way or factorial analyses of variances with or without repeated measures) as appropriate. Values in graphs represent the mean ± s.e.m. Sample sizes are indicated in the results or figure legends. The results were considered statistically significant if *p* < 0.05, but trends *p* < 0.1 are also shown.

All analysis of behavior testing was performed blind to treatment conditions.

## Results

Behavioral phenotypes of both males and females from the F0 generation was measured, and differences in aggression, anxiety, and depressive tendencies were observed between PPS and control groups (Supplementary Figs. 2–5). Given that we observed these marked differences in both the F0 males and females, we next assessed the behavioral phenotype of the cross-fostered offspring in the following categories.

### Aggressive behavior

#### Play behavior

In juveniles, aggressive temperament, as measured by TAB was enhanced. This increase in aggressive temperament (2-way ANOVA on square-root transformed values to obtain normality, *n* = 17, 16, 16, and 15 males respectively) during home cage play behavior (Fig. 2A) was seen as a result of both prenatal (*F*_(1,60)_ = 9.1, *p* = 0.0038) and postnatal (*F*_(1,60)_ = 6.0, *p* = 0.018) PPS dam influences (interaction, *F*_(1,60)_ = 0.056, *p* = 0.8140).

#### Cohabitation and resident intruder

In adulthood, aggression in males was assessed by two behavioral paradigms—cohabitation with partner females, and the resident-intruder test. Adult males reared by PPS male-paired dams exhibited increased aggressive behavior towards females during cohabitation (Fig. 2B). Two-way ANOVA (on square-root transformed data to obtain normality, *n* = 8 males per group) revealed significant postnatal effect on the percentage of offensive behavior relative to other social actions, including hetero-grooming, sniffing, and mounting *F*_(1,28)_ = 10.6; *p* = 0.003 (interaction, *F*_(1,28)_ = 0.03, *p* = 0.87 and prenatal, *F*_(1,28)_ = 0.62, *p* = 0.44).

Consistent with these results, a significant postnatal effect on the percentage of offensive behavior was also observed towards an intruder male in the resident-intruder test (Fig. 2C; Two-way ANOVA, *n* = 8 males per group, postnatal, *F*_(1,28)_ = 4.8; *p* = 0.037; prenatal, *F*_(1,28)_ = 0.12, *p* = 0.73; interaction, *F*_(1,28)_ = 0.31, *p* = 0.58). This increase in aggression was also reflected in the total frequency of offensive behaviors, i.e. number of attack, keep down, offensive upright and lateral threat (postnatal, *F*_(1,28)_ = 5.50; *p* = 0.026; prenatal, *F*_(1,28)_ = 0.44, *p* = 0.51; interaction, *F*_(1,28)_ = 0.43, *p* = 0.52; Fig. 2D).

### Social behavior towards non-threatening juvenile

#### Social preference

A reduced social preference (Two-way ANOVA, *n* = 8 males per group) was due to decreased exploration of the juvenile in the stressed animals, with both prenatal (*F*_(1,28)_ = 7.72, *p* = 0.0096) and postnatal influences (Fig. 3A; *F*_(1,28)_ = 17.97, *p* < 0.001; interaction, *F*_(1,28)_ = 0.21, *p* = 0.65). There were no significant differences in time spent exploring the object among the groups (Fig. 3B). Similarly, while all groups showed a preference for the juvenile over the object (one-sample t-tests: Ctrl-Ctrl [*t*_(7)_ = 20.7; *p* = 0.000], Ctrl-PPS [*t*_(7)_ = 14.0; *p* = 0.000], PPS-Ctrl [*t*_(7)_ = 7.7; *p* < 0.001], PPS-PPS [*t*_(7)_ = 5.7; *p* = 0.001]), nevertheless, a lower inclination for social contact (i.e. relatively more social avoidance; Fig. 3B) was observed when considering the juvenile exploration percentage (expressed as the time spent exploring the juvenile over total exploration), significantly reduced both by being born to or reared by PPS male-paired dams (postnatal, *F*_(1,28)_ = 17.99, *p* = 0.0002; prenatal, *F*_(1,28)_ = 7.73, *p* = 0.01; interaction, *F*_(1,28)_ = 0.21, *p* = 0.65).

#### Social memory

Following the social preference test, animals were tested in the same three-chambered apparatus with simultaneous exposure to two juveniles. All groups discriminated between novel and familiar (Fig. 3C); one-sample t-tests: [Ctrl–Ctrl, *t*_(7)_ = 4.3; *p* = 0.004; Ctrl-PPS, *t*_(7)_ = 2.4; *p* = 0.048; PPS-Ctrl, *t*_(7)_ = 3.90; *p* = 0.006; PPS–PPS, *t*_(7)_ = 9.79; *p* < 0.001]). Yet, and in contrast to the apparent reduced propensity for social investigation described above (Fig. 3B), social memory appeared enhanced, and by postnatal condition only (greater novel juvenile exploration percentage i.e. time spent exploring novel juvenile/total exploration) (Fig. 3C, Two-way ANOVA, *n* = 8 males per group, postnatal, *F*_(1,28)_ = 8.6, *p* = 0.007; prenatal, *F*_(1,28)_ = 0.01, *p* = 0.92; interaction, *F*_(1,28)_ = 2.58, *p* = 0.12). This enhanced preference for social novelty emerged as a result of a significant postnatal decrease in exploration of the familiar juvenile (*F*_(1,28)_ = 8.47, *p* = 0.007) without differences in novel juvenile or the total exploration (*p* > 0.3) (Fig. 3D).

### Non-social coping behavior

#### Shock prod

In the shock prod test, active vs passive coping styles were assessed. The offspring in a postnatal PPS male-paired female environment exhibited less immobility in response to the shock (Fig. 4A, Two-way ANOVA on square-root transformed data to obtain normality, *n* = 8 males per group, postnatal effect, *F*_(1,28)_ = 8.2, *p* = 0.008; prenatal effect, *F*_(1,28)_ = 3.1, *p* = 0.089; interaction, *F*_(1,28)_ = 0.09, *p* = 0.77). The reduction in immobility in this condition reflected increased rearing (postnatal, *F*_(1,28)_ = 4.3, *p* = 0.049; prenatal, *F*_(1,28)_ = 0.73, *p* = 0.40; interaction, *F*_(1,28)_ = 1.04, *p* = 0.32), yet for the key measures of active coping, investigating and burying the shock prod (probe explore), the postnatal condition increase did not reach statistical significance (Postnatal, *F*_(1,28)_ = 3.09; *p* = 0.089; prenatal, *F*_(1,28)_ = 0.97, *p* = 0.33; interaction, *F*_(1,28)_ = 0.50, *p* = 0.49) by animals reared by PPS paired females. Furthermore, no effects were seen when considering these behaviors separately either (Fig. 4B).

#### Forced swim

In the forced swim test, more active coping was expressed due to postnatal exposure to PPS male-paired dam, according to both swimming extent (Fig. 4C, effect on Day 1, two-way ANOVA, *n* = 8 males per group, postnatal, *F*_(1,28)_ = 9.5, *p* = 0.005; prenatal, *F*_(1,28)_ = 1.85, *p* = 0.19; interaction, *F*_(1,28)_ = 1.32, *p* = 0.26) and increased latency to float (Fig. 4D, postnatal, *F*_(1,28)_ = 4.5, *p* = 0.044; prenatal, *F*_(1,28)_ = 0.22, *p* = 0.64; interaction, *F*_(1,28)_ = 0.52, *p* = 0.48).

## Discussion

Here, we have investigated to what extent the early postnatal maternal environment contributes to transgenerational changes in aggressive behaviors in the offspring of peripubertal stressed male rats. We also looked at how these postnatal influences (maternal environment) affect the social and coping behaviors of these offspring. This question is relevant, given that the females submitted to intimate partner violence are known to develop PTSD and depressive-like behaviors [38], similar to the dams submitted to the rodent model of intimate partner violence studied here [23, 25].

Using an early adoption approach, our findings allow the disentangling of transgenerational effects induced by the prenatal environment (i.e., gestational influences) from those induced by changes in maternal behavior (as a consequence of exposure to aggressive males). Such a factor has been previously shown to be capable of shaping offspring behaviors, for example, coping to stress and social behaviors [39–41].

The first goal of our study was to extensively characterize the behavioral phenotype that arises in the offspring of peripubertally stressed males, reared by their birth mothers (i.e., PPS male-paired dams) and their foster mothers (i.e., control male-paired dams) in order to determine the relative impact of prenatal vs postnatal influences. We observed that the subjects born to and raised by the PPS male-paired dams exhibited more aggression, both towards unfamiliar males, and towards females, right from the beginning of cohabitation. Increased number and faster attacks during play behavior also confirm the aggressive phenotype of these animals. They also exhibited decreased sociability. Social memory also revealed a strong postnatal effect of reduced exploration of the familiar juvenile, indicating better memory. Furthermore, these offspring exhibited more active forms of coping, as evidenced through increased swimming in the forced swim task and increased rearing in the shock prod test. These active coping mechanisms may reflect changes in risk assessment behavior in these subjects. Overall, the contrasting pattern of observed negative social outcomes (reduced sociability and increased aggression) coupled with the improved social memory as well as increased active coping (notably in forced swim test) suggests that early life postnatal stress can cause animals to be become resilient and more capable of coping with stressful situations in later stages of life [42].

Therefore, our results regarding the social memory and depressive-like outcomes do not support the “twohit” stress model that states that experiencing stressors at different developmental time periods enhances stress-negative outcomes in brain function and behavior. Our findings align well with a number of recent studies in the literature showing that effects of stressors experienced at different life periods are not necessarily additive, but may in fact lead to protective effects. Indeed, several rodent studies have shown that postnatal stress followed by adverse experiences either during adolescence [43, 44] or at adulthood [45] protected against a number of stress-related phenotypes (e.g., cognitive deficits, depressive- and anxiety-like behaviors).

Furthermore, the present study identifies the importance of the postnatal environment (maternal effects) in mediating some of the offspring behavior by studying the maternal behavior of the dams paired with the PPS males. However, we should also acknowledge that the small sample size may have deterred from revealing potential milder influences exerted by the prenatal environment, and future experiments are warranted to fully address the scope of prenatal effects. While typically measured maternal behaviors appeared normal, replicating our previous finding [23], here we observed some subtle behavioral differences in PPS male-paired dams, for example a change in their mouthing behavior and pup retrieval (Supplementary Fig. 3D). The depressive-like behavior was replicated in the dams, and further testing revealed the detrimental behavioral and physiological effect of cohabitation with stressed males as evidenced through an increased negative interpretation predisposition (Supplementary Fig. 4). Although we cannot establish causality here, such maternal changes may influence the offspring behavioral outcomes, as observed between child oppositional behavior and maternal depression [46]. For a detailed description of behavioral tests performed on PPS male-paired dams, refer to the Supplementary material.

In addition, other postnatal factors beyond maternal behavior, such as maternal lactocrine [47, 48] and microbiota might be considered [49]. Moreover, postnatal sibling interactions might also play a role. Here, post weaning, groups were segregated into pairs of individuals from the same condition. Therefore, it is plausible that interactions that were moderated prior weaning were subsequently amplified, contributing to the observed pattern wherein the prenatal influences seen in adolescence gave way to predominance of postnatal influences at adulthood. This hypothesis is further supported by the interaction of both prenatal and postnatal effects observed during juvenile play behavior.

Although it would be speculative to propose any specific mechanisms mediating the impact of prenatal and/or postnatal influences described here, one of the mechanisms that have been particularly implicated in the long-term impact of prenatal and postnatal stress exposures is the modulation of the hypothalamus-pituitary-adrenal (HPA) axis. However, whereas prenatal stress exposure generally leads to increases in circulating corticosterone levels [50], postnatal effects tend to be more varied and highly depending on the developmental stage when stress exposure takes place, as well as the context in which it happens [51]. Our results suggest that latent changes induced by the prenatal adverse environment are amenable of being modified by postnatal influences. Both prenatal and early postnatal adversity can have a strong impact on the development of neural circuits that regulate stress responsiveness [52], and modulation of expression levels of glucocorticoid receptors have been implicated on the impact of postnatal stressful influences in adult social behavior [53]. Furthermore, both types of experiences are known to lead to epigenetic changes [54] and alterations in brain microstructure [55].

Multigenerational programming effects are a complex interplay of several mechanisms. Based on the results described above, we contend that maternal care and temperament play a salient role in the development of a phenotype in offspring that was similarly expressed paternally. The results of the cross-fostering manipulation emphasize that the behavioral outcome of epigenetic modifications in the germline of PPS males, if any, may be superseded by appropriate postnatal maternal care.

Interestingly, many preclinical studies have reported that effects caused by early trauma in offspring (deleterious or advantageous) can be reversed by providing favorable conditions later in life (Environmental Enrichment or EE) [56–58]. These findings suggest that postnatal influences can normalize or aggravate behaviors altered due to prenatal stress, regardless of the genetic predisposition of the offspring [59, 60]. Furthermore, similar observations have also been reported in humans, wherein the Callous-Unemotional (CU) traits (developmental precursor to psychopathy) in children who were institutionalized were prevented by providing high-quality foster care [61–63].

Altogether, our study highlights the importance of timely interventions to facilitate the healthy development of children born to disturbed and violent families. In the future, it will be important to extend this type of preclinical studies to females, and to study other endpoints, such as cognitive function. Early life parent assistance has been proposed as a means of improving care for the long-term benefit of the offspring [64, 65]. Interpretive bias modification is also of potential relevance [66]. Thus, the present results support the notion that, in spite of prenatal influences and potential epigenetic modifications, invaluable benefits may be conferred by investment in the postnatal environment, especially in early life [67], to pre-empt persistent psychopathology.

## Supplementary information

Supplemental Material
