# Supplementary material for: Early life adoption shows rearing environment supersedes transgenerational effects of paternal stress on aggressive temperament in the offspring
Source: Transl Psychiatry. 2021 Oct 16;11:533. doi: 10.1038/s41398-021-01659-2 (PMC8520526; doi:10.1038/s41398-021-01659-2)
Supplement: Supplementary file 1 — Supplemental Material [file 41398_2021_1659_MOESM1_ESM.pdf]

SUPPLEMENTARY INFORMATION

S1. METHODS:

S1.1 Maternal Behavior – There are two commonly used methods of examining maternal behavior in rodents – periodic/spot-check observations of the subject's undisturbed behavior with the litter, and continuous observation, ranging from 5 min, to 4 hours (Lonstein and Fleming, 2001). We employed both approaches, performing spot checks on P4 and P16 and continuous checks on P6 and P8. For periodic checks, each cage was observed thrice for 1 min, with 30 min separation between each observation. Continuous observations were performed on P6 without disturbing the cage and P8 following maternal separation. We quantified the following behavior – actively hovering around the pups, including kurtosis (arched-back posture), supine (lying on its back or side with nipples exposed), prone (lying on the litter with no limb support), grooming pups, mouthing, nest construction, self-grooming, sleeping separately, eating/drinking water and ambulation (Lonstein and Fleming, 2001).

1.1.1 Maternal Separation: On postnatal day 8, pups and mothers were separated for 2 min. For each trial, the dams were kept in their home cage, while all the pups were placed together in a beaker. The dam was subsequently removed, and the pups were scattered across the home cage. The dam was returned to the cage, and the maternal behavior was video recorded for 15 min. The latency to retrieve each pup back to the nest was noted.

1.1.2 Nest Assessment: Nests were assessed one day before the day of birth and on postnatal days 5, 8 and 16. Without disturbing the cage, the nest was scored by the following parameters (Lonstein and Fleming, 2001): 0=no nest, paper strips still scattered over entire floor of the cage; 1=poor nest, not all paper strips are used and the nest is flat; 2=fair nest, all paper is used but the nest is flat; 3=good nest, all paper is used and the nest has relatively low walls (<5 cm); 4=excellent nest, all paper is used and the nest has relatively high walls (>5 cm).

S1.2 Judgement bias – An ecological test of response to ambiguous stimuli was adapted (Boleij et al., 2012). During training and test trials pre-weighed 500g almond pieces were presented on a small Petri dish (diameter 5.5 cm). The odors were spread on a tissue in a total amount of 6μl, which was positioned in the Petri dish. The Petri dish with the filter paper was covered by a lid with several holes to let the odors diffuse through the top. Animals were habituated to eating a piece of almond in their home cages for three consecutive days before the training. Testing and training was done between 10:00 am and 4:00 pm. During all trials, the home-cage was placed on a stand, with the lid on. Animals were removed from the cage, the petri dish with an almond placed on the lid was placed at one end of the cage, and the animal was replaced facing the other direction of the cage. The entire duration of training and testing was 5 days, with the first 3 days being positive trials, day 4 being a negative trial, and day 5 being the test trial. During training in a positive trial, the odor was presented with a normal tasting almond piece and in a negative trial a different odor was presented with a bitter tasting almond piece. In the test trials, both odors were presented together with a normal tasting almond piece. Almond pieces were made bitter by dipping them in a 180 mmol quinine solution (Sigma–Aldrich) and drying them overnight. The odors used were rose and green apple and were counterbalanced within a group for positive and negative trials. Observations for the latency to approach, pick, start, and finish eating the almond were taken for all 5 days. Each trial lasted till either the almond was finished or for an upper limit of 5 min. Response bias was investigated by statistically comparing latencies to pick and start eating the almond piece in the positive trials with that in the test trials, and the ratio between the time taken on the 5th day to that on the 3rd day was determined to be an appropriate measure of a judgment bias.

S1.3 Organs and body weight – The females were weighed every week to identify differences in their weight gain during and after cohabitation. Organs were extracted from rats taken from the home cage four days after the last test. Animals were decapitated at basal levels. Trunk blood was collected in heparin-coated tubes, centrifuged for 4 min at 10 000 rpm at 4°C, and the supernatant plasma was stored with Aprotinin (Sigma-Aldrich) at -20°C. The brains were frozen in isopentane on dry ice, wrapped with foil, and stored at -80°C. Adrenals were extracted and weighed separately.

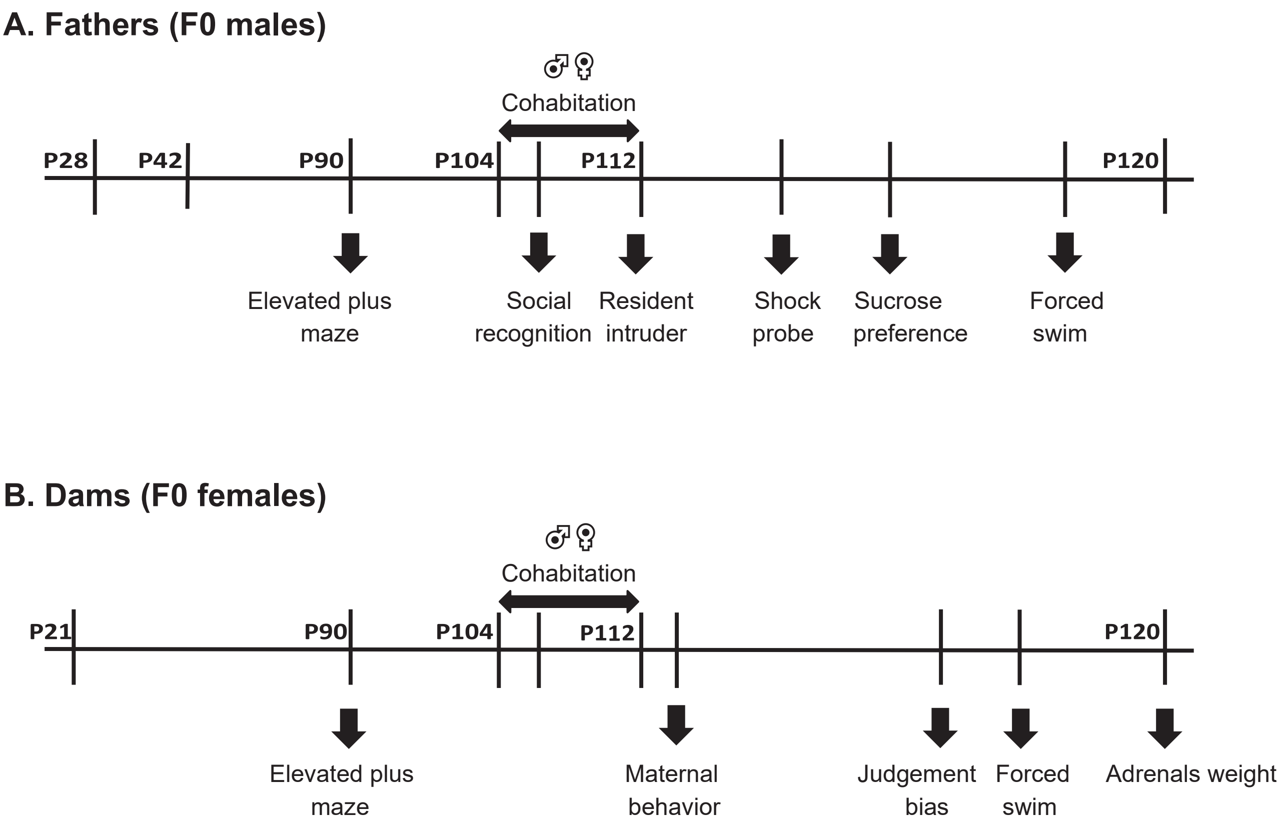

Supplementary figure 1. Battery of behavioral tests done on F0 males and females (males - control and PPS; females - control paired and PPS paired).

(**A**) Experimental timeline of fathers (F0 males). (**B**) Experimental timeline of dams (F0 females).

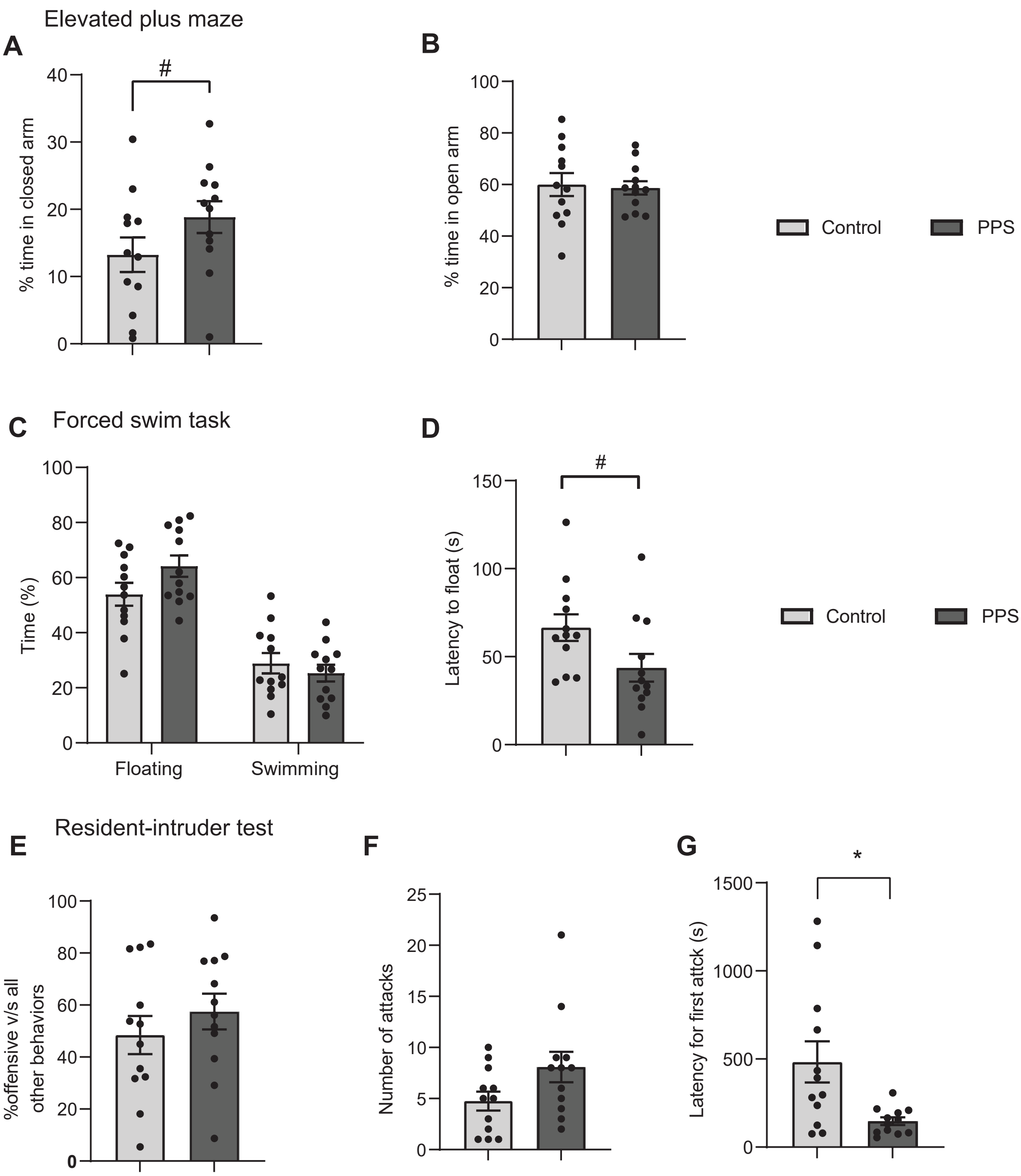

Supplementary figure 2. F0 PPS-males displayed increased aggression, and tendecies towards anxiety and depressive-like behavior.

(**A, B**) Time spent in the open and closed arm on the elevated plus maze. PPS males had a tendency to spend more time in the closed arm (Wilcoxon signed rank test, n = 12 males per groups, *p(closed, open) = 0.09, 0.63*). (**C**) There were no difference between the groups in duration spent swimming or floating in the forced swim task (Wilcoxon signed rank test, n = 12 males per groups, *p(floating, swimming) = 0.31, 0.64*). (**D**) PPS males displayed a tendency to start floating sooner in the forced swim task (Wilcoxon signed rank test, n = 12 males per groups, Z = 1.72, *p = 0.08*). (**E**) Percentage of offensive behavior in comparison to other social actions, i.e., heterogrooming and sniffing does not reveal differences between the groups (Wilcoxon signed rank test, n = 12 males per groups, *p = 0.43*). (**F**) Total number of attacks on the intruder did not differ between groups (Wilcoxon signed rank test, n = 12 males per groups, *p = 0.12*). (**G**) The latency for first attack was lower in PPS males (Wilcoxon signed rank test, n = 12 males per groups, Z = 2.11, *p = 0.03*). The results are the mean ± s.e.m. PPS: Peripubertally stressed rats. # *p <0.1, * P <0.05*

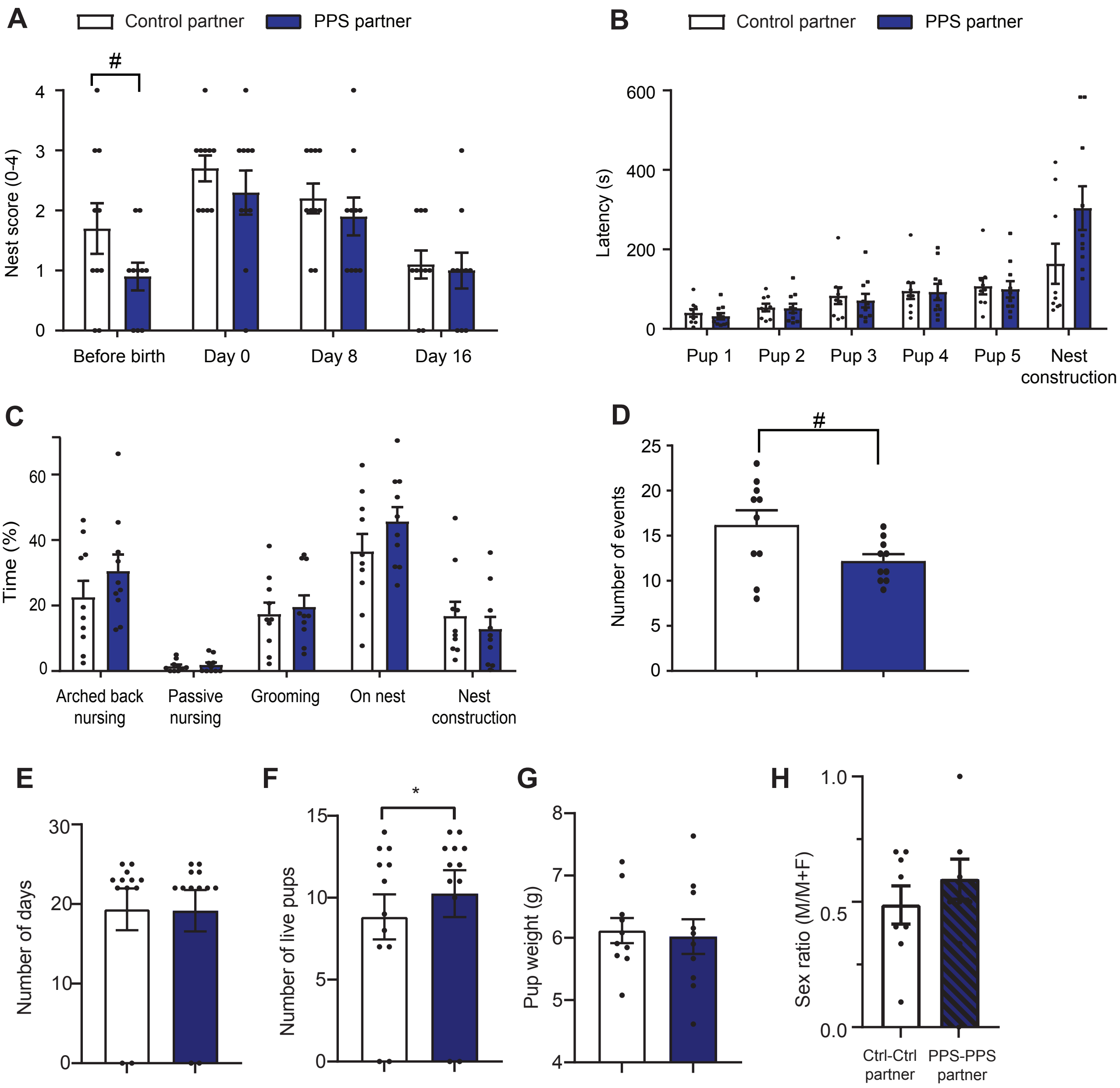

Supplementary figure 3. Assessment of maternal behavior revealed minor differences between control-male paired and PPS-male paired dams.

(**A**) There was a tendency for control-male paired dams to build better nests before the birth of the pups. This effect went away after the pups were born (independent samples Wilcoxon signed rank test, n = 10 females per group, *p(before birth, day0, day8, day16) = 0.05, 0.32, 0.52, 0.71*).(**B**) In the maternal separation test, performed on postnatal day 8, there was no difference in the latency to retrieve the first five pups back to the nest (independent samples Wilcoxon signed rank test, n = 10 females per group, *p(pup1, pup2, pup3, pup4, pup5, nest construction) = 0.86, 0.86, 0.83, 0.95, 0.77, 0.17*). (**C**) No differences were observed between the two groups for the time spent in any of the nursing positions, licking-grooming or general contact with the pups (independent samples Wilcoxon signed rank test, n = 10 females per group, *p(arched back, passive, grooming, on nest, nest construction*)= 0.21, 0.78, 0.14, 0.17, 0.86). (**D**) There was a tendency for the frequency of mouthing (carrying pups around the cage) to be higher in control male paired dams than in PPS paired dams (independent samples Wilcoxon signed rank test, n = 10 females per group, Z = 1.68, *p = 0.09*). (**E**) Number of days between start of cohabitation and birth of pups for F0 females did not differ by condition (independent samples Wilcoxon signed rank test, n = 10 females per group, *p = 0.48).* (**F**) PPS male-paired dams had significantly larger litter sizes compared to control (independent samples Wilcoxon signed rank test, n = 10 females per group, Z = -2.12, *p = 0.034).* Only pups that were born alive were counted. (**G**) Average weight of newborn pups revealed no differences in the weight of pups (independent samples Wilcoxon signed rank test, n = 10 females per group, *p = 0.11).* (**H**) Because pups were culled to litters of 10 and cross fostered immediately after birth for the F0 females, we did not estimate the sex ratio of pups in this generation. However, we measured the sex ratio of the pups from the F1 dams (i.e., F2 generation), and no differences were observed between the groups (independent samples Wilcoxon signed rank test, n = 8 females per group, *p = 0.75*). The results are the mean ± s.e.m. Ctrl: Control rats, PPS: Peripubertally stressed rats. # *p<0.1*, * *p<0.05*

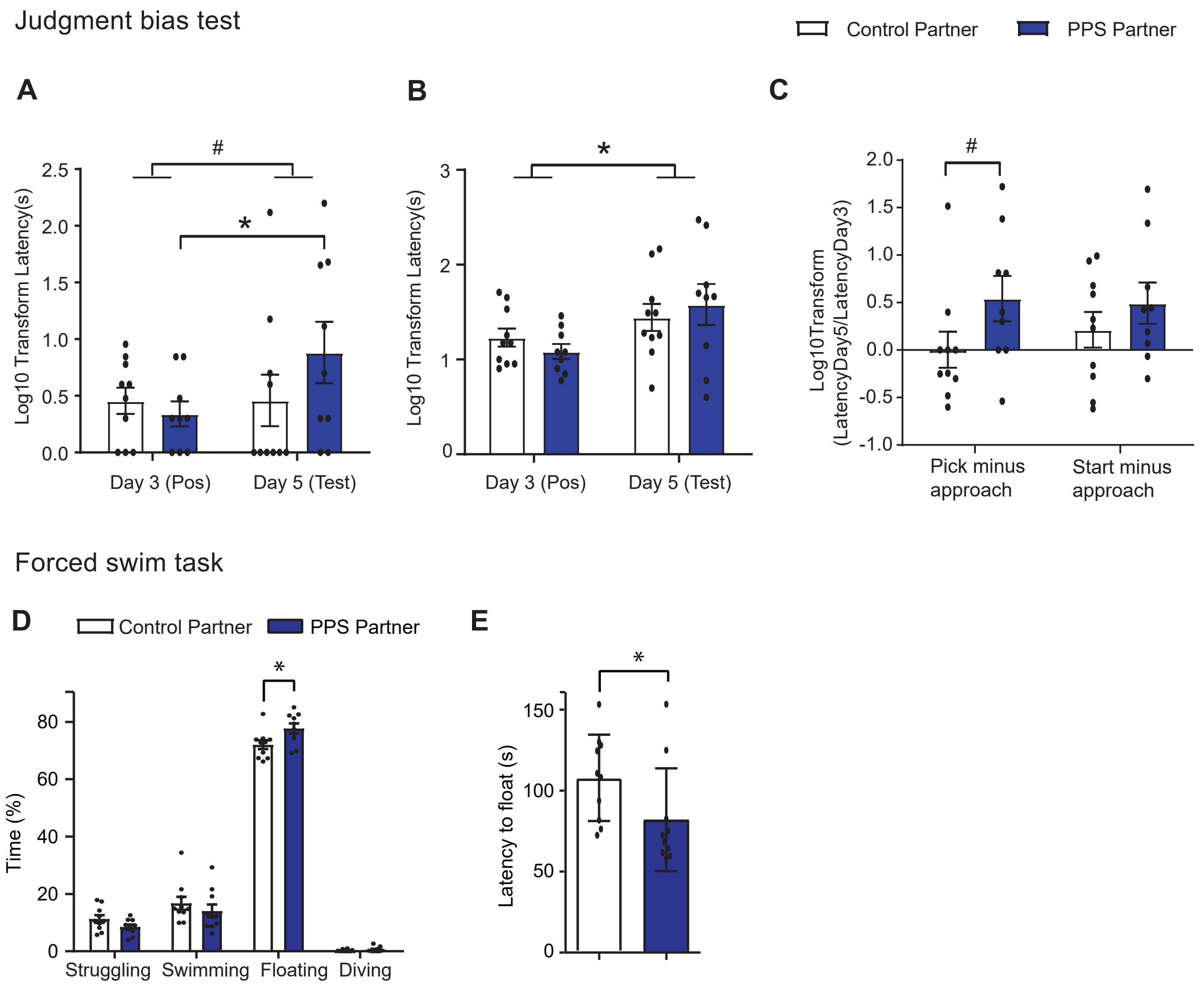

Supplementary figure 4. PPS-male paired dams were observed to have an increased negative bias and displayed depressive-like tendencies.

(**A**) Latency (pick minus approach) to pick the almond after the first approach shows trends for the day effect (n = 10 females per group, $F(1,17)=3.26$, $p = 0.089$) and stress x day interaction ($F(1,17)=3.18$, $p = 0.092$). Dams paired with stressed rats take significantly longer on day 5 than on day 3 (n = 10 & 9 females per group, $p=.024$). (**B**) Latency (start minus approach) to start eating the almond after the first approach, with a significant day effect (n = 10 & 9 females per group, $F(1,17)=6.116$, $p=0.024$). (**C**) The individual ratios of the time taken on day 5 and day 3 to pick after approach and start eating after approach show a trend for the hesitancy to pick up the almond in females paired with stressed males ($t(17) = -1.78$; $p = 0.092$). Only dams with pups were tested in this experiment. (**D**) PPS male-paired dams spent more time floating (independent samples Wilcoxon signed rank test, n = 12 females per group, *p(struggling, swimming, floating, diving) = 0.14,0.24,0.03, 0.14)* as compared to control male-paired dams. (**E**) There was a decreased latency to start floating in PPS male-paired dams (independent samples Wilcoxon signed rank test, n = 12 females per group, Z = 2.04, $p = 0.04$). The results are the mean ± s.e.m. PPS: Peripubertally stressed rats. # $p < 0.1$,* $p < 0.05$

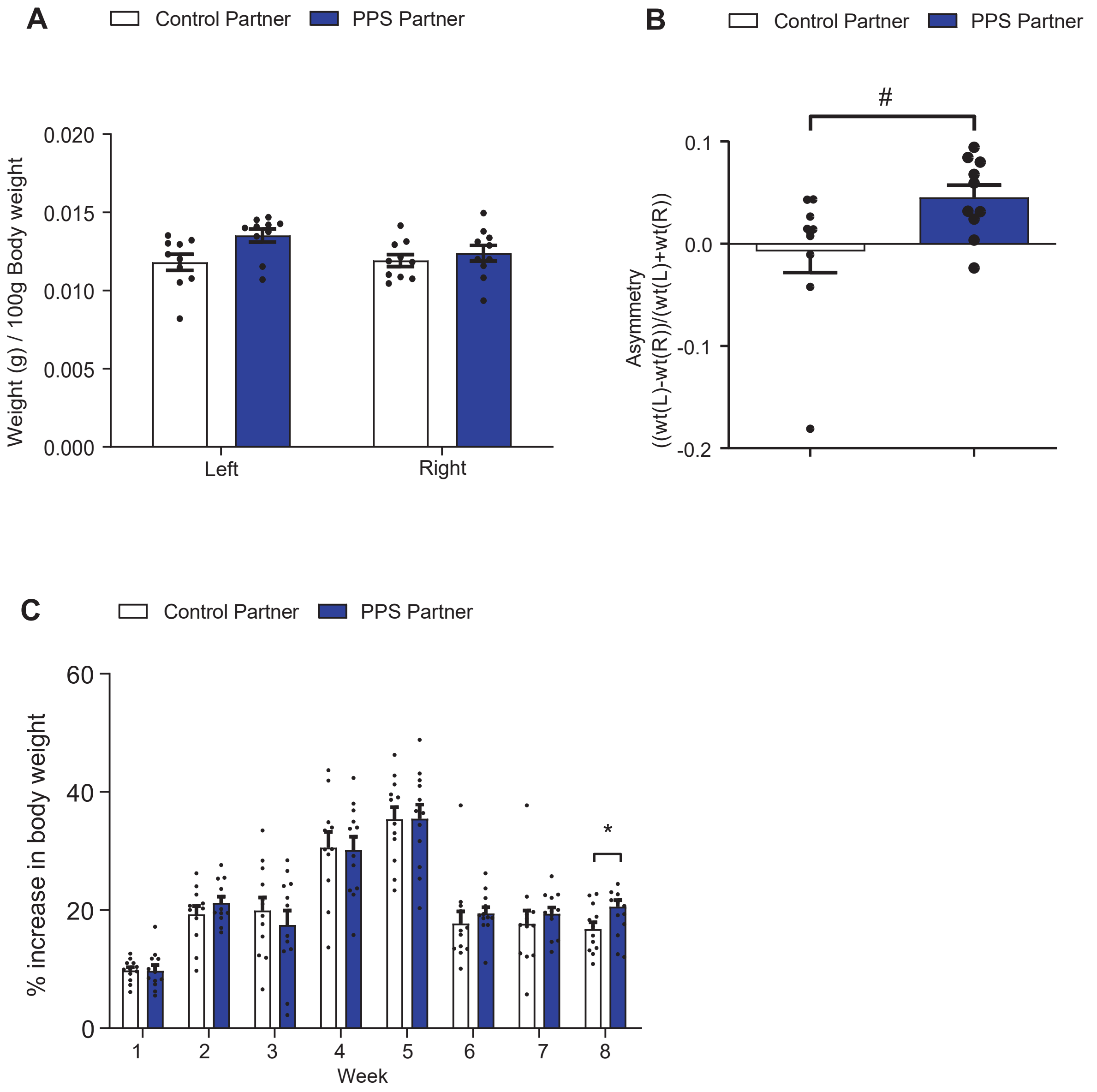

Supplementary figure 5. MInor changes in adrenal and body weight was observed in PPS-male paired dams.

(**A**) Weight of left and right adrenal glands, with no changes in the average weight between groups (Wilcoxon signed rank test, n = 12 females per group, *p(left, right) = 0.13, 0.94*). t(18) = 0.479; p = 0.018). (**B**) Lateralization of the adrenals indicating an increased left-based asymmetry in dams paired with PPS males (Wilcoxon signed rank test, n = 12 females per group, *p = 0.07*). (**C**) Increase in body weight per week since cohabitation indicated no differences in the body weights between the two groups for all the weeks, except for week 8 (Wilcoxon signed rank test, n = 12 females per group, *p(1,2,3,4,5,6,7,8) = 0.75, 0.27, 0.43, 0.87, 0.75, 0.14, 0.34, 0.022*). The results are the mean ± s.e.m. PPS: Peripubertally stressed rats*. # p<0.1, * p <0.05*
